# Nanostructured Geometries Strongly Affect Fouling of Carbon Electrodes

**DOI:** 10.1021/acsomega.1c03666

**Published:** 2021-09-29

**Authors:** Ayesha Kousar, Emilia Peltola, Tomi Laurila

**Affiliations:** † Department of Electrical Engineering and Automation, School of Electrical Engineering, Aalto University, 02150 Espoo, Finland; ‡ Department of Chemistry and Materials Science, School of Chemical Engineering, Aalto University, 02150 Espoo, Finland

## Abstract

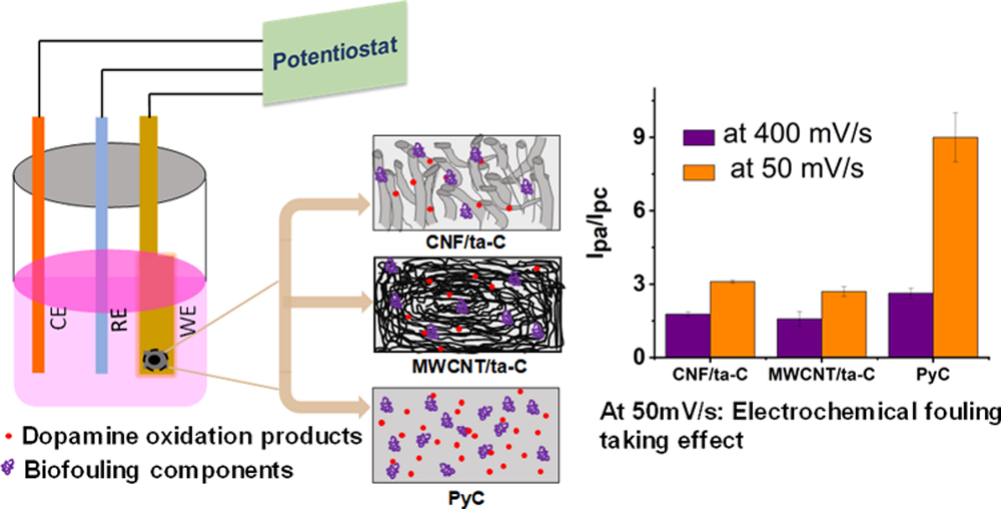

Electrode fouling is a major factor that compromises the performance of biosensors in *in vivo* usage. It can be roughly classified into (i) electrochemical fouling, caused by the analyte and its reaction products, and (ii) biofouling, caused by proteins and other species in the measurement environment. Here, we examined the effect of electrochemical fouling [in phosphate buffer saline (PBS)], biofouling [in cell-culture media (F12-K) with and without proteins], and their combination on the redox reactions occurring on carbon-based electrodes possessing distinct morphologies and surface chemistry. The effect of biofouling on the electrochemistry of an outer sphere redox probe, [Ru(NH_3_)_6_]^3+^, was negligible. On the other hand, fouling had a marked effect on the electrochemistry of an inner sphere redox probe, dopamine (DA). We observed that the surface geometry played a major role in the extent of fouling. The effect of biofouling on DA electrochemistry was the worst on planar pyrolytic carbon, whereas the multiwalled carbon nanotube/tetrahedral amorphous carbon (MWCNT/ta-C), possessing spaghetti-like morphology, and carbon nanofiber (CNF/ta-C) electrodes were much less seriously affected. The blockage of the adsorption sites for DA by proteins and other components of biological media and electrochemical fouling components (byproducts of DA oxidation) caused rapid surface poisoning. PBS washing for 10 consecutive cycles at 50 mV/s did not improve the electrode performance, except for CNF/ta-C, which performed better after PBS washing. Overall, this study emphasizes the combined effect of biological and electrochemical fouling to be critical for the evaluation of the functionality of a sensor. Thus, electrodes possessing composite nanostructures showed less surface fouling in comparison to those possessing planar geometry.

## Introduction

Surface fouling of electrode materials is known to cause loss of sensitivity and reproducibility of electrochemical sensing *in vivo*.^1,2^ The fouling components form an impermeable layer on the electrode surface, blocking the electrode surface, which affects the electron-transfer reactions of the analyte molecules. Surface fouling can be categorized into two classes: (i) biofouling (passive fouling), which is caused by components in the biological environment, such as proteins and lipids, and (ii) electrochemical fouling (active fouling), in which the byproduct(s) of the analyte molecules’ redox reaction block the electrode surface.^3^ However, these two phenomena occur simultaneously while detecting dopamine (DA) in biological systems. The extent of surface fouling is controlled by several factors. The tendency of biofouling is associated with the hydrophobicity/hydrophilicity of the electrode surface.^4^ Hydrophobic surfaces are more susceptible to biofouling as compared to hydrophilic surfaces. However, proteins can be adsorbed through both hydrophobic and hydrophilic interactions.^5,6^ Electrode surface roughness is another factor that can enhance the anti-biofouling properties of electrodes. In fact, biofouling studies have been a major focus of analytical electrochemists studying *in vivo* biosensing.^7^ However, it is equally important to evaluate the electrode surfaces for both biofouling and electrochemical fouling as the degree of electrode passivation can be altered by the presence of both types of fouling. Additionally, electrodes may also exhibit differing tendencies toward electrochemical and biofouling.^8^ For example, Harreither et al. reported that carbon nanotube (CNT) fiber electrodes fouled by bovine serum albumin (BSA) cause a reduction in polydopamine layer formation on the electrode surface. They argued that amine sulfur moieties in BSA compete with amine functionalities for nucleophilic binding of catecholamine in the critical step of DA fouling (i.e., nucleophilic binding to catechol).^9^

Reduced surface fouling has been accomplished through the incorporation of functionalized polymer films onto electrode surfaces.^10,11^ For example, Liu et al. functionalized polyethylenedioxythiophene (PEDOT) with phosphorylcholine (PC-PEDOT) and electropolymerized it on a carbon fiber microelectrode (CFME) surface, imparting zwitterionic functionality to the electrode. Besides reducing biofouling, the PEDOT-PC/CFME electrode also did not exhibit loss of a DA signal even after 2 h of electrode implantation in the rat brain.^12^ Similarly, Singh et al. used coatings of Nafion, base-hydrolyzed cellulose acetate (BCA), and fibronectin on CFME to study selectivity, sensitivity, and resistance to fouling. BCA and fibronectin-coated CFME showed resistance to fouling but at the expense of reduced sensitivity or selectivity of cationic neurotransmitters.^13^ In addition, electrode coating with negatively charged polymers, such as Nafion and oPPy, prevents the adsorption of redox products and biomolecules, leading to lower electrode fouling.^14^ Polymer layers can cause unnecessary electrostatic interactions with changes in the external environment, reducing biofouling while affecting DA adsorption.^15^ Moreover, the formation of thick polymer layers on the electrode surface can affect the temporal resolution of the sensor, which can lead to slower response rates and ineffectiveness for long-term *in vivo* sensing.^13^ Thus, modulation of the surface properties of carbon materials, such as CNTs, carbon nanofibers (CNFs), highly oriented pyrolytic graphite, and boron-doped diamond, can be an effective approach for future research. For instance, Weese et al. analyzed the effect of defect sites on CFME, pristine CNTs, and functionalized CNTs on biofouling and chemical fouling in serotonin and reported that these sites exhibit different effects on the extent of biofouling and electrochemical fouling of CFME and CNTs.^8^ To address this challenge, the morphology and surface chemistry of carbon electrodes should be tuned to regulate surface fouling resistance. However, the surface characteristics must be carefully considered when constructing a sensor that is least susceptible or unsusceptible to fouling.

In this study, we select three carbon materials with different surface structures: CNFs with a Ni catalyst on tetrahedral amorphous carbon (ta-C) film, multiwalled CNTs (MWCNTs) on ta-C, and planar pyrolytic carbon (PyC). The CNF structure contains vertically aligned nanofibers of approximately 1 μm length and 75 nm diameter, whereas MWCNTs form a uniform network of approximately 10 μm long intertwined fibers with an average bundle diameter of 5–10 nm. PyC belongs to the class of nanographitic thin-film materials containing numerous edge plane sites. CNF and MWCNT surfaces are considerably rougher than the PyC surface. All these materials have been reported to exhibit excellent DA detection performance in phosphate buffer saline (PBS) in our previous studies.^16−20^ In this study, we evaluated the electrochemical performance of PyC and hybrid carbon-based electrodes in different complex media to study the effects of biofouling and electrochemical fouling on DA detection. A biological medium (F12-K) with proteins [15% horse serum (HS) and 2.5% fetal bovine serum (FBS)], F12-K without proteins, and PBS were used to study and compare the effects of biofouling and electrochemical fouling on carbon-based electrodes. The F12-K medium contained l-arginine, pyruvic acid, glutamine, and small concentrations of various amino acids, lipids, vitamins, and salts. The F12-K + protein medium contained the same components as those of the F12-K medium with the addition of various proteins, including albumin, fibrinogen, globulin, hemoglobin, fibronectin, agglutinins, and vitronectin and various enzymes, carbohydrates, and hormones. These media were chosen as they contained the nutrients required for the cells to grow and accurately mimic the biological environment. This helped us estimate how the real biological environment affects the sensor performance. Biofouling and electrochemical fouling are correlated with surface properties, such as roughness, morphology, and hydrophobicity/hydrophilicity. The kinetics of an outer sphere redox (OSR) probe, [Ru(NH_3_)_6_]^3+^, were investigated to study the effects of the different media and biofouling on simple electron-transfer kinetics. Furthermore, a known inner sphere redox (ISR) probe, DA, was used to assess the effect of combined bio- and electrochemical fouling on the electron-transfer kinetics.

## Results and Discussion

### Structural Characterization

**Figure 1:**
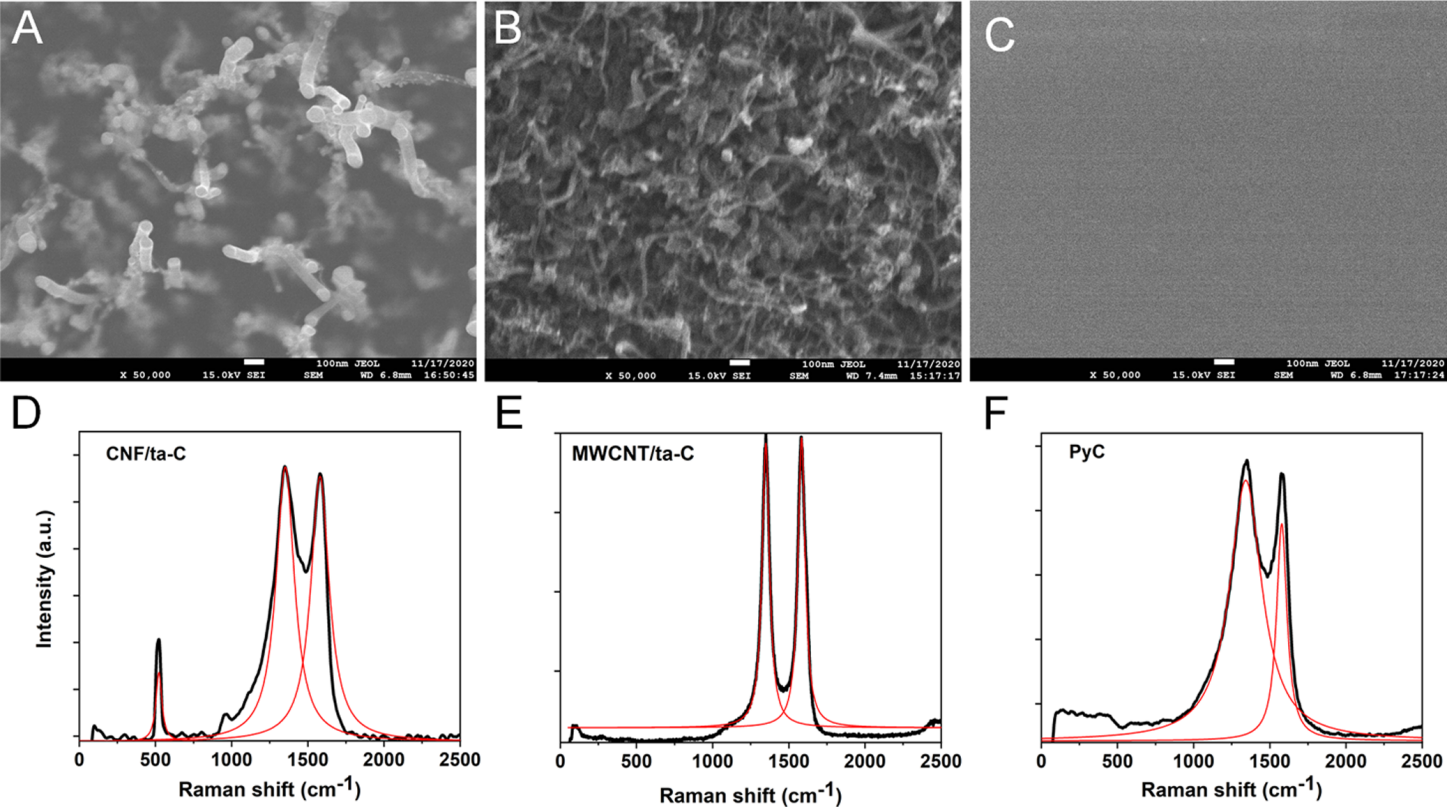
SEM images of (A) CNF/ta-C, (B) MWCNT/ta-C, and (C) PyC. Raman spectra of (D) CNF/ta-C, (E) MWCNT/ta-C,^18^ and (F) PyC.

The susceptibility of biofouling and electrochemical fouling was anticipated to significantly differ depending on the changes in the geometrical and chemical properties of the electrodes. Furthermore, the presence of FBS and various salts in the biological medium with and without proteins was expected to exhibit different adsorption behaviors on different electrode surfaces. Thus, to gain insights into the biofouling and electrochemical fouling patterns exhibited by different types of CNTs, we used carbon-based surfaces with drastically different surface morphologies. In addition, scanning electron microscopy (SEM) was performed to study the topography of the electrode surfaces (Figure 1). The surface properties evaluated previously through X-ray adsorption spectroscopy,^21,22^ Raman spectroscopy, and contact angle measurements are presented in Table 1.

**Table 1:** Morphology and Surface Chemistry Trends of the Electrode Materials^a^

				surface chemistry trends					
geometry and electrode type	morphology	fiber dimensions	roughness estimation (PyC as the planar reference)^b^	sp^2^	sp^3^	sp	COOH andketone/aldehyde	*I*_d_/*I*_g_ ratio	contact angle (hydrophobicity/hydrophilicity)
forest-like (CNF/ta-C)	vertically aligned fibers with Ni particles attached^c^	1 μm length, 50–75 nm diameter, and 200–500 nm spacing between fibers	18 X	√			√ (considerable)	1.1	θ_adv_ = 86 ± 2° θ_rec_ = 32 ± 2° (weakly hydrophilic)
spaghetti-like (MWCNT/ta-C)	porous network of intertwined and curved fibers	10 μM length and 5–10 nm bundle diameter	100 X	√			√	1-highly defective	θ_adv_ ≈ 86–120° θ_rec_ < 40°^d^ (hydrophobic)
planar (PyC)	flat surface (RMS 1.2 ± 0.6 nm)^16^		1 X	√	√	√	√ (negligible)	3.17	θ_adv_ = 60 ± 1° θ_rec_ = 22 ± 2° (moderately hydrophilic)

a Surface chemistry studies (XAS and Raman spectroscopy) and morphology have been previously reported: CNF/ta-C and MWCNT/ta-C^19−21,23^ and PyC.^16,22^

b Roughness estimation was performed by double-layer capacitance studies.

c Some chunks of fibers and smaller structures between the main fibers are also observed.

d Contact angles were not stable for MWCNT/ta-C due to highly non-uniform surface.

The table presents information about the geometry, morphology, surface chemistry, and wettability of the samples. Figure 1 shows the SEM images and Raman spectra, which demonstrate the morphology and defect trends of the electrodes, respectively. The information presented in Figures 1 and S1 and Table 1 is summarized as follows. We have presented detailed X-ray absorption spectroscopy (XAS) data in the previous publications.^21,22^ However, the numbers obtained in those previous papers are not directly comparable due to differences in data fitting. Therefore, we present a qualitative comparison of the trends observed in surface chemistry. The surfaces of CNF/ta-C electrodes, which exhibited forest-like geometry, were significantly rough, defective, and weakly hydrophilic, possessing high sp^2^ content with several functional groups, such as COOH and OH. The MWCNT/ta-C electrode surfaces, possessing a porous network of fibers arranged in a spaghetti-like fashion, were highly uneven, defective, and hydrophobic. The moderately hydrophilic planar PyC surface contained a high carbon content with negligible functionalities and a high *I*_d_/*I*_g_ ratio, indicating the presence of the sp^3^-bonded carbon and highly reactive sp-type carbon. The modulation of free surface energies could play an important role in controlling the fouling of the electrodes.^24−26^ However, due to the non-uniformity of MWCNT/ta-C and CNF/ta-C, we could not get reliable contact angle measurements for several liquids, thus limiting the possibility to study the surface energies.

The use of carbon nanomaterials in different forms enhances the roughness of electrodes without increasing their size and can promote DA adsorption.^27^ Porous and array-like nanostructures such as MWCNTs, carbon nanospikes, and carbon nanopipettes may entrap the DA molecules leading to thin-layer electrochemistry.^28,29^ Formation of the thin-film mass-transfer regime on the electrode surface affects the redox reaction of the analyte by enhancing the cyclization of catecholamines and can also improve the temporal resolution.^30−32^ Similarly, electrochemical fouling caused by DA also gets affected by the surface structure as CNT fiber (CNTF) microelectrodes possessing irregular structure reported to show three times lesser fouling than CF microelectrodes with homogeneous structures.^33^ The contribution of thin-film liquid layer formation to electrochemical detection of DA due to porous structures of MWCNT/ta-C and electrochemical sensing properties of CNF/ta-C and PyC has been described in detail in our previous articles including also the geometric effects on fouling.^16,19,23^ A substantial amount of the literature is available describing the thin-liquid-layer trapping effects of different geometries on the detection of DA. However, mechanistic details about the effect of both electrochemical and biofouling conditions on the sensing ability of these geometries have been rarely studied. We have correlated with the presence/absence of nanostructures and current contribution by adsorption and diffusion processes on electrode surfaces in the presence of electrochemical and biofouling conditions.

### Background Measurements

Blank measurements were performed in all media (Figure S2). To study the effects of different constituents of the biological media on the electrode surface, 50 cycles in F12-K with and without proteins at 50 mV/s were performed. CNF/ta-C and MWCNT/ta-C did not show a significant decrease in the background current with the number of cycles in any media. MWCNTs showed higher the double-layer charging current for the F12-K + protein medium than the F12-K medium. In contrast, CNF showed higher background current for F12-K than that for F12-K + protein, indicating different interactions of the constituents of the two media with both electrode surfaces due to different geometries. PyC showed low background current for both media, where the cyclic voltammogram (CV) for F12-K + protein showed a continuous drop in current during cycling (Figure S2). The pseudocapacitance (*C*_dl_) of the electrical double layers of all electrodes in the different media was calculated from the CVs recorded at different scan rates (10, 50, 100, and 400 mV/s). The differences between the anodic and cathodic currents (μA) were plotted against the scan rate (*v*), and the slope of the plot was divided by the geometric area of the corresponding electrode following the Δ*i* = 2 × *C* × *v* equation. MWCNT/ta-C exhibited significantly larger *C*_dl_ than other electrodes because of its porous structure [one-way analysis of variance (ANOVA), *p* value < 0.0001, *n* = 3]. As shown in Table 2, for all electrodes, *C*_dl_ was the highest in PBS among the three media. CNF/ta-C achieved a *C*_dl_ value of 528 ± 73 μF/cm^2^ in PBS, which decreased to 354 ± 20 μF/cm^2^ in the case of F12-K and further to 225 ± 56 μF/cm^2^ in the presence of proteins indicating significant changes with the change in media (one-way ANOVA, *p* value < 0.005). In the case of MWCNT/ta-C, *C*_dl_ in PBS was calculated as 2852 ± 95 μF/cm^2^, which decreased to 1270 ± 164 μF/cm^2^ for F12-K. However, for F12-K + protein, *C*_dl_ did not undergo a significant decrease in comparison to PBS and was calculated as 2597 ± 199 μF/cm^2^ (unpaired *t*-test, *p* value > 0.05) (Table 2). In the case of PyC, *C*_dl_ had values of 28 ± 5, 20 ± 4, and 18 ± 4 μF/cm^2^ in PBS, F12-K, and F12-K + protein, respectively, showing insignificant differences with the change in media (one-way ANOVA, *p* value > 0.05). The lack of nanostructures made the PyC surface smoother than the surfaces of the other electrodes used in the study, which is attributed to its smallest electrochemical area. In all cases, faradic (surface) reactions contributed significantly to pseudocapacitance, especially at lower scan speeds.

**Table 2:** Double-Layer Capacitance of Electrodes Calculated from the Slope of Δ*i* versus *v* at Different Scan Rates (10, 50, 100, and 400 mV/s)

medium	CNF/ta-C μF/cm^2^	MWCNT/ta-C μF/cm^2^	PyC μF/cm^2^
F12-K	354 ± 20	1270 ± 164	20 ± 4
F12-K + proteins	225 ± 56	2597 ± 199	18 ± 4
PBS	528 ± 73	2852 ± 95	28 ± 5

To estimate the roughness of the electrode surfaces, we divided the measured *C*_dl_ values of MWCNT/ta-C and CNF/ta-C with the *C*_dl_ value of PyC, which was considered flat to obtain the indicative surface roughness enhancement factor in PBS.^34^ The CNF/ta-C electrode exhibited an approximately 18 times larger surface area than PyC, whereas MWCNT/ta-C exhibited hundred times larger surface area than PyC. Note also that these materials did not indicate a clear double-layer region, which indicates the effect of pseudocapacitance caused by the faradic reactions occurring on the electrode surface in the presence of different functionalities, as stated above (Table 2).

To study the effect of biofouling and electron-transfer kinetics of the redox reaction occurring on the electrode surfaces in different media, CVs for an OSR probe, [Ru(NH_3_)_6_]^3+^, and an ISR probe, DA, were recorded.

### OSR System

**Figure 2:**
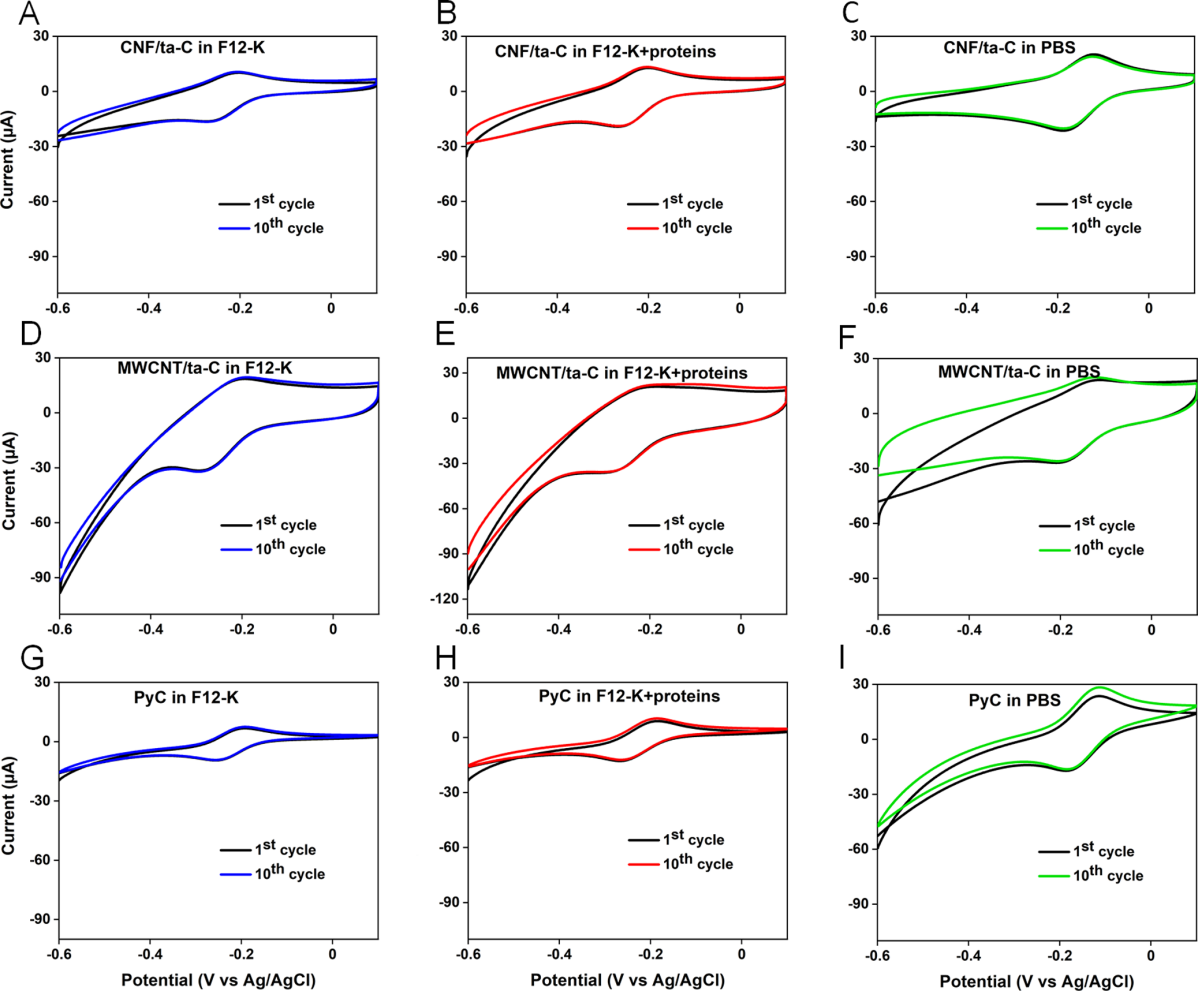
CVs of [Ru(NH_3_)_6_]^3+^ for (A–C) CNF/ta-C, (D–F) MWCNT/ta-C, and (G– I) PyC showing 1st (black) and 10th cycles (blue: F12-K, red: F12-K + protein, and green: PBS) at 50 mV/s. The concentration of [Ru(NH_3_)_6_]^3+^ is 1 mM.

None of the three electrodes in the three media showed a noticeable current drop from the 1st to 10th cycle for [Ru(NH_3_)_6_]^3+^ at 50 mV/s (Figure 2). As the electroactive area of the MWCNT/ta-C electrode was much larger than those of CNF/ta-C and PyC electrodes, the peak current exhibited in the CVs is not proportionally higher for MWCNTs in comparison to the other two electrodes in the case of all media. To evaluate the contribution of semi-infinite diffusion and thin-liquid-layer effects on mass transfer, the CVs were recorded at different scan rates (Figure S3). The extent to which a reaction is administered by diffusion and thin-layer electrochemistry can be predicted as 0.5 or 1, respectively, by the slope of log *I*_pa_ versus log *v*.^35^ The CNF/ta-C electrode showed slopes of 0.64, 0.56, and 0.57 in F12-K, F12-K + protein, and PBS, respectively, indicating that semi-infinite diffusion is the predominant factor in mass transfer. For the MWCNT/ta-C electrode, the slope ranged from 0.59 to 0.63 in all three media, indicating that semi-infinite diffusion is responsible for the majority of the current contribution. Similarly, the PyC electrode showed slopes of 0.43, 0.57, and 0.43 in F12-K, F12-K + protein, and PBS, respectively, predicting semi-infinite mass transfer (Table 3).

**Table 3:** Oxidation Potential (*E*_pa_), Peak Separation (Δ*E*_p_), Oxidation Current (*I*_pa_), Ratio of Oxidation and Reduction Current (*I*_pa_/*I*_pc_), Heterogeneous Electron-Transfer Rate Constant (*k*_0_), and Matsuda–Ayabe Parameter (Λ) of Different Electrodes for [Ru(NH_3_)_6_]^3+^ in Different Media

			Δ*E*_p_ (mV)				*k*_0_		Matsuda–Ayabe parameter		
electrode	electrolyte	*E*_pa_(mV)	50 mV/s	400 mV/s	*I*_pa_(μA)	*I*_pa_/*I*_pc_	50 mV/s	400 mV/s	50 mV/s	400 mV/s	slope (log *I*_pa_ vs log v)
CNF/ta-C	F12-K	–193 ± 10	62 ± 2	86 ± 7	13 ± 1	1.4 ± 0.3	0.095 ± 0.007	0.0167 ± 0.002	27.4	1.54	0.64 ± 0.01
	F12-K + protein	–200 ± 4	62 ± 1	80 ± 2	14 ± 2	1.4 ± 0.1	0.092 ± 0.03	0.021 ± 0.001	26.4	1.96	0.560 ± 0.004
	PBS	–122 ± 5	63 ± 0	106 ± 11	14 ± 1	0.80 ± 0.06	0.075 ± 0.01	0.0092 ± 0.0008	21.6	0.85	0.57 ± 0.02
MWCNT/ta-C	F12-K	–206 ± 14	68 ± 3	126 ± 10	20 ± 7	1.6 ± 0.4	0.036 ± 0.02	0.0058 ± 0.0001	10.4	0.53	0.63 ± 0.02
	F12-K + protein	–208 ± 9	74 ± 3	131 ± 14	16 ± 6	2.2 ± 0.2	0.014 ± 0.001	0.0054 ± 0.0006	4.1	0.50	0.59 ± 0.01
	PBS	–121 ± 10	76 ± 4	124 ± 11	17 ± 3	1.80 ± 0.09	0.01 ± 0.00	0.0063 ± 0.0006	2.8	0.58	0.60 ± 0.02
PyC	F12-K	–198 ± 3	62 ± 4	72 ± 2	9.8 ± 3	1.9 ± 0.07	0.09 ± 0.06	0.019 ± 0.002	28	1.83	0.43 ± 0.06
	F12-K + protein	–190 ± 4	66 ± 7	91 ± 19	9.5 ± 1.7	1.30 ± 0.05	0.07 ± 0.04	0.009 ± 0.003	21.7	0.83	0.57 ± 0.02
	PBS	–111 ± 4	61 ± 5	82 ± 6	10 ± 3.7	1.40 ± 0.04	0.09 ± 0.06	0.020 ± 0.003	28.3	1.88	0.43 ± 0.01

The changes in the media did not cause significant changes in the oxidation current (*I*_pa_) for all electrodes (one-way ANOVA, *p* value > 0.05, *n* = 3), whereas the reduction current (*I*_pc_) showed changes that varied among the three electrodes. The oxidation potential (*E*_pa_) did not vary significantly for different electrodes in each medium (one-way ANOVA, *p* value > 0.05) (Table 3, Figures 2 and S3). Peak-to-peak separation (Δ*E*_p_) was observed for two scan rates (50 and 400 mV/s) and used as a measure to predict the electron-transfer kinetics. To further evaluate the kinetics of the electrochemical reaction, we calculated the heterogeneous electron-transfer rate constant (*k*_0_) and the Matsuda–Ayabe parameter (Λ) at 50 and 400 mV/s, respectively. Matsuda and Ayabe defined “Λ” as a parameter that quantifies the reversibility of a reaction and is related to the heterogeneous electron-transfer rate constant, as follows
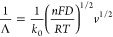


A reaction is assumed to be kinetically reversible in the case of Λ > 15, quasi reversible for 15 > Λ < 0.001, and irreversible for Λ < 0.001. For all electrodes, lower reversibility and sluggish kinetics were obtained at the higher scan rate (400 mV/s) as compared to 50 mV/s. CNF/ta-C showed nearly ideal kinetics in all three media with Δ*E*_p_ ≈ 62–63 mV at 50 mV/s. The value of *k*_0_ for CNF/ta-C was slightly higher in F12-K than that in F12-K + protein (Table 3). However, the opposite trend was observed at 400 mV/s, where the value of *k*_0_ in F12-K + protein was higher than that in F12-K. The CNF/ta-C electrode achieved Λ value ranging from 26 to 27 in F12-K and F12-K + proteins and 21.6 in PBS, which evidenced a kinetically reversible reaction at a slow scan rate. In the case of the MWCNT/ta-C electrode at 50 mV/s, [Ru(NH_3_)_6_]^3+^ showed faster electron-transfer kinetics in F12-K (Δ*E*_p_ ≈ 68 mV) compared to F12-K + protein (Δ*E*_p_ ≈ 74 mV), which indicates an enhancement of *k*_0_ in F12-K. The trend observed for the MWCNT/ta-C electrode remained the same at higher scan rates as well, where Δ*E*_p_ increased more in F12-K + protein than that in F12-K (Figure S3, Table 3). The MWCNT/ta-C electrode exhibited quasi reversible behavior in all three media, as demonstrated by the Λ value in the range 4–10 in the biological media and 2.8 in PBS. At both scan rates, PyC showed slightly higher electron-transfer kinetics in F12-K than that in F12-K + protein. However, with a further increase in the scan rate, Δ*E*_p_ remained almost constant in both F12-K and F12-K + protein (Figure S3). In all three media, a kinetically reversible reaction was observed at 50 mV/s with Λ ranging from 21 to 28 (Table 3). These results indicate that the proteins did not exhibit visible effects on the electron-transfer kinetics of [Ru(NH_3_)_6_]^3+^ on CNF/ta-C and PyC; however, MWCNT/ta-C showed slower electron transfer in F12-K + protein than that in F12-K.

### ISR System

The effect of biofouling and electrochemical fouling on the kinetics of DA, a known surface-sensitive probe, was studied on all three electrode surfaces using a biological medium (F12-K) with and without proteins as well as in PBS. CVs were recorded at different scan rates to estimate the contribution of diffusion and adsorption to the total current (Figure S4). 100 μM concentration of DA has been used in all experiments to quantify the electrochemical fouling, which is higher than the physiologically relevant DA range (50 nM to 1 μM).^36^ Although a lower level of electrochemical fouling is expected to occur from the physiological concentration of DA, the higher concentration of DA was used to expedite the electrochemical fouling process. This gives us insights into the long-term behavior of the electrode for electrochemical sensing and consequently about the lifetime of the electrodes. Furthermore, the presence of biofouling components may also affect the rate of electrochemical fouling.^9^

**Figure 3:**
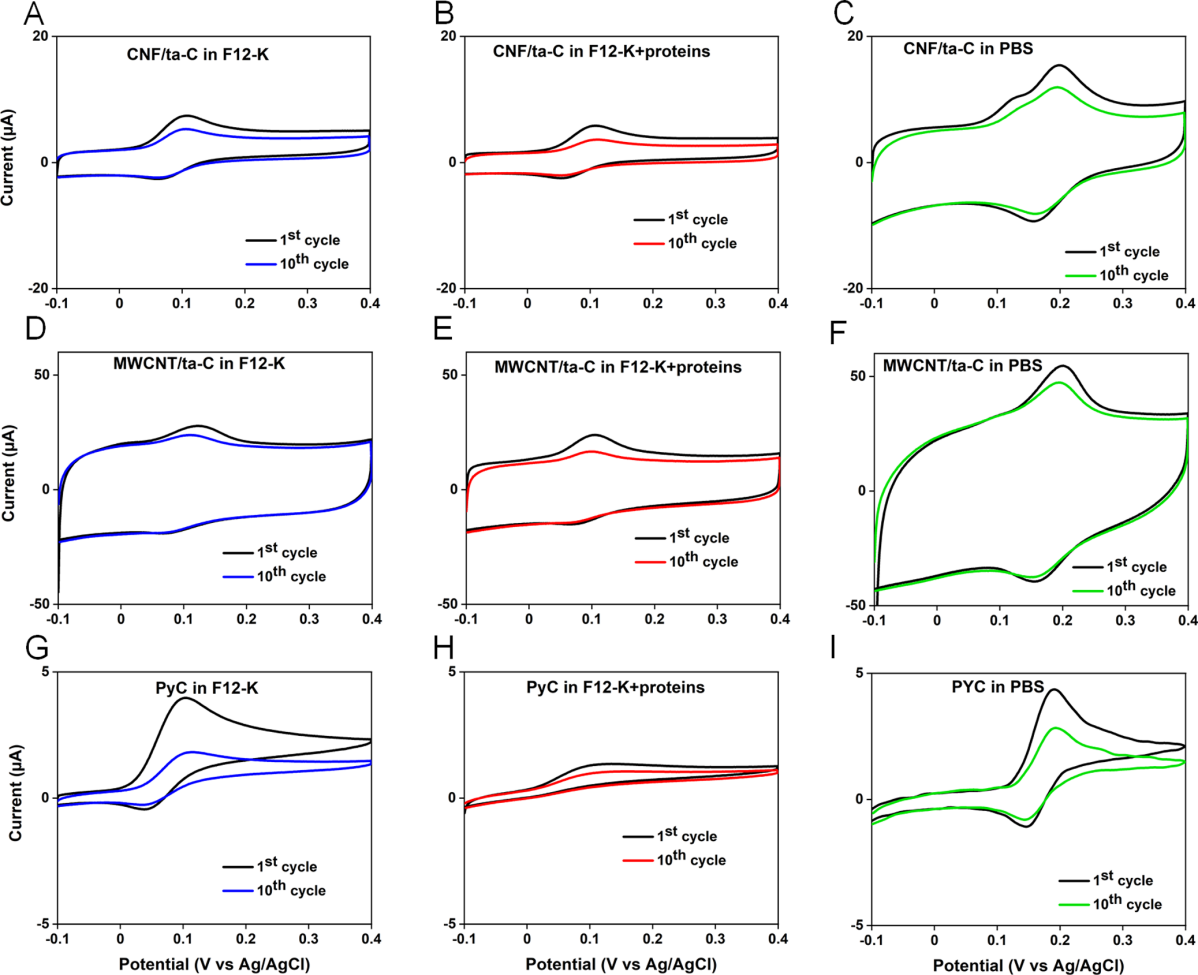
CVs of the DA reaction for (A–C) CNF/ta-C, (D–F) MWCNT/ta-C, and (G–I) PyC showing 1st and 10th cycles in different media at 50 mV/s.

**Figure 4:**
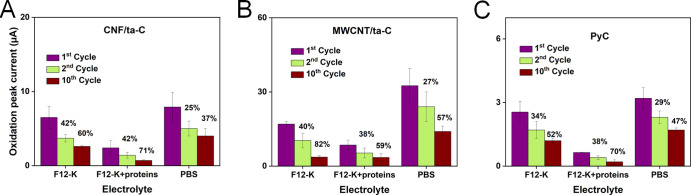
Current drop (also indicated in percentage) for (A) CNF/ta-C, (B) MWCNT/ta-C, and (C) PyC in the 2nd and 10th cycles as compared to the 1st cycle in different media. The DA concentration is 100 μM, and the scan rate is 50 mV/s.

The percentage current drop from the 1st to 10th cycle and Δ*E*_p_ changes in PBS and the biologically mimicking solutions were studied to estimate the interactions and effects of the media components on DA kinetics over the electrode surfaces (Figures 3 and 4).

The oxidation peak current was significantly high for MWCNT/ta-C in all media (one-way ANOVA, *p* value < 0.0005 *n* = 2–4) due to the several times higher surface roughness of MWCNT/ta-C compared to the those of CNF and PyC (Figure 4). In PBS, the MWCNT/ta-C electrode achieved an *I*_pa_ value of 32.5 ± 7.5 μA, whereas CNF/ta-C and PyC achieved values of 8 ± 2 and 3.5 ± 0.5 μA, respectively. In F12-K, the *I*_pa_ values were 17 ± 1 μA for MWCNT/ta-C, 6.5 ± 1.5 μA for CNF/ta-C, and 2.6 ± 0.4 μA for PyC. In F12-K + protein, MWCNT/ta-C achieved an *I*_pa_ value of 8.5 ± 2 μA, whereas CNF/ta-C and PyC achieved values of 2.4 ± 1 and 0.64 ± 0.1 μA, respectively. The significant reduction in the *I*_pa_ value in F12-K + protein in comparison to F12-K (unpaired *t*-test, *p* value < 0.05) indicates that the proteins decreased the electroactive surface area of all three electrodes (Table 4).

**Table 4:** Electrochemistry of DA in Biological Media in Comparison to PBS

			Δ*E*_p_ (mV)			*I*_pa_/*I*_pc_		
electrode	electrolyte	*E*_pa_ (mV)	50 mV/s	400 mV/s	*I*_pa_ (μA)	50 mV/s	400 mV/s	slope (log *I*_pa_ vs log *v*)
CNF/ta-C	F12-K	100 ± 5	41 ± 6	67 ± 7	6.5 ± 1.5	1.60 ± 0.04	1.38 ± 0.25	0.72 ± 0.05
	F12-K + proteins	96 ± 18	58 ± 21	103 ± 22	2.4 ± 1.0	3.10 ± 0.06	1.77 ± 0.10	0.60 ± 0.01
	PBS	191 ± 4	35 ± 5	90 ± 6	8 ± 2	1.5 ± 0.2	1.16 ± 0.09	0.80 ± 0.04
MWCNT/ta-C	F12-K	118 ± 7	63 ± 18	150 ± 22	17 ± 1	2 ± 0.3	1.28 ± 0.05	0.80 ± 0.07
	F12-K + proteins	76 ± 5	37 ± 5	88 ± 12	8.5 ± 2	2.7 ± 0.2	1.58 ± 0.36	0.82 ± 0.02
	PBS	203 ± 4	43 ± 3	198 ± 19	32.5 ± 7.5	1.560 ± 0.005	1.18 ± 0.05	0.9 ± 0.1
PyC	F12-K	107 ± 6	65 ± 3	93 ± 3	2.6 ± 0.4	4 ± 1	1.43 ± 0.02	0.50 ± 0.01
	F12-K + proteins	179 ± 15	146 ± 17	250 ± 33	0.6 ± 0.1	9 ± 1	2.63 ± 0.24	0.40 ± 0.02
	PBS	196 ± 9	60 ± 22	128 ± 19	3.5 ± 0.5	3 ± 0.2	1.31 ± 0.02	0.59 ± 0.09

### Electrochemical Fouling

Electrochemical fouling was estimated in PBS for all three electrodes. CNF/ta-C and MWCNT/ta-C exhibited 37 and 57% oxidation current drop from the 1st to 10th cycle, respectively, and PyC exhibited 47% drop (Figure 4). However, no notable Δ*E*_p_ changes were observed. This suggests that for all electrodes in PBS, the electrochemically active surface area decreased, and the reaction kinetics were not significantly influenced from the 1st to 10th cycles. At 50 mV/s, the *I*_pa_/*I*_pc_ values were highest for PyC (3 ± 0.2), indicating a significant reduction in *I*_pc_ than those for CNF/ta-C (1.5 ± 0.2) (unpaired *t*-test, *p* value < 0.005, *n* = 3) and MWCNT/ta-C (1.560 ± 0.005) (unpaired *t*-test, *p* value < 0.005). This suggests lesser interaction of dopamine-*o*-quinone (DAQ) with the PyC surface as compared to the other two electrodes, which could be because the PyC surface possesses fewer functional groups, such as COOH.^37^ At 400 mV/s, all electrodes achieved lower *I*_pa_/*I*_pc_ values than those observed at 50 mV/s and the difference in these values *I*_pa_/*I*_pc_ among the electrodes decreased (Table 4). This indicates that at a slower scan rate, the chemical reaction step after the oxidation of DA, which converts DAQ to dopaminechrome (DAC), has sufficient time to occur, subsequently removing DAQ from the system and lowering the value of *I*_pc_. In contrast, at higher scan rates, the chemical step is partially outrun (Figure S4). The difference between the *I*_pa_/*I*_pc_ values at 50 and 400 mV/s was the smallest for CNF/ta-C, slightly higher for MWCNT/ta-C, and remarkably high for PyC. Similarly, the Δ*E*_p_ values were the smallest for CNF/ta-C (35 ± 5 mV), followed by MWCNT/ta-C (43 ± 3 mV) and PyC (60 ± 22) (Table 4), which indicates the fast reaction kinetics of DA on CNF/ta-C.

The DA reaction produced another peak pair around −0.3 and −0.4 V in the cyclic voltammetry performed in a wider potential window, which represents the redox reaction of leucodopaminechrome (LDAC) and DAC in addition to the DA and DAQ redox pair on electrode surfaces (Figure S5). CNF/ta-C and MWCNT/ta-C demonstrated prominent peaks associated with the LDAC ⇋ DAC reaction at all scan rates. In contrast, PyC demonstrated small and broad peaks, which were associated with the second redox couple (LDAC ⇋ DAC). The change in Δ*E*_p_ as a function of the scan rate was smaller for CNF/ta-C than those for the other two electrodes, indicating that the forest-like geometry influences the reaction kinetics. As shown in Table 3, the slope of log *I*_pa_ versus log *v* predominantly predicted the adsorption-controlled reaction mechanism by MWCNT/ta-C (0.9 ± 0.1), mixed control (adsorption and diffusion) by CNF/ta-C (0.8 ± 0.04), and predominance of diffusion-controlled electrochemistry by PyC (0.59 ± 0.09). Although we cannot differentiate between thin-liquid-layer formation and adsorption behavior without proper washout experiments, the lack of any indication of drastic thin-liquid-layer formation with an OSR probe indicates the effect of adsorption on these nanostructured surfaces. These results indicate that composite carbon nanostructures are favorable materials for DA detection as electrochemical fouling is slower on these electrode surfaces. However, in the presence of proteins and other components such as salts, vitamins, and amino acids, the separate estimation of electrochemical fouling and biofouling is difficult. Next, we attempt to correlate the phenomena to the best possible extent.

### Biofouling

With an increase in Δ*E*_p_, in F12-K, the peak current for CNF/ta-C dropped to approximately 60% in the 10th cycle, whereas in F12-K + protein, the current dropped to 71% (Table 4, Figure S4). These qualitative results indicate that the proteins decrease the electrochemically active area and hinder the overall rate of the reaction. The ratio of decrease in the reduction peak also increased in the presence of proteins in all media. The reaction appeared to be under mixed adsorption and diffusion control (slope log *I*_pa_ vs log *v* = 0.72 ± 0.05) in F12-K, whereas in the presence of proteins, diffusion (slope log *I*_pa_ vs log *v* = 0.60 ± 0.01) was a predominantly controlling step, indicating significantly higher blockage of adsorption sites, with the proteins lowering the contribution of adsorption to the total current. The MWCNT/ta-C electrode showed interesting electrochemistry in the biological media. The MWCNT/ta-C electrode exhibited mixed control, with predominance of adsorption-controlled reactions, in all media, with log *I*_pa_ versus log *v* slope = 0.80 ± 0.07 and 0.82 ± 0.02 for F12-K and F12-K + protein, respectively, unlike the other electrodes used in this study. The DA oxidation peak shifted to the cathodic direction in F12-K + protein compared to F12-K and PBS (Table 4). Approximately 59% current dropped from 1st to 10th cycle in F12-K + protein, whereas in F12-K, approximately 82% current drop was unexpectedly observed (Figure 4).

The estimation of the log *I*_pa_ versus log *v* slope on the PyC showed diffusion-controlled reaction in F12-K (slope = 0.50 ± 0.01) and predominantly diffusion control in F12-K + protein (slope = 0.40 ± 0.02). Oxidation current was low in the presence of proteins, with very broad oxidation peak and almost no reduction peak. Approximately, 70% *I*_pa_ dropped in F12-K + protein, whereas 52% *I*_pa_ dropped in F12-K, indicating a faster blocking of an electrochemically active area in the presence of proteins. Sluggish kinetics were observed in F12-K + protein, as indicated by Δ*E*_p_ = 146 ± 17 mV, whereas slow kinetics were observed in F12-K, as indicated by Δ*E*_p_ = 65 ± 3.

### Combination of Biofouling and Electrochemical Fouling

To study the combined effect of electrochemical fouling and biofouling, we examined the *I*_pa_/*I*_pc_ values of DA and DAQ at 50 and 400 mV/s in the biological media. As shown in Table 4, at 50 mV/s, the CNF/ta-C electrode achieved significantly higher *I*_pa_/*I*_pc_ values for DA/DAQ in F12-K protein (3.10 ± 0.06) than that in F12-K (1.6 ± 0.04) (unpaired *t*-test, *p* value < 0.001, *n* = 3), indicating a lack of DOQ near the electrode surface (or passivated surface), which is responsible for a smaller reduction peak. The MWCNT/ta-C electrode achieved *I*_pa_/*I*_pc_ values of 2 ± 0.3 and 2.7 ± 0.2 in F12-K and F12-K + protein (significant difference, unpaired *t*-test, *p* value < 0.05), respectively. PyC exhibited significantly high *I*_pa_/*I*_pc_ values of 4 ± 1 in F12-K and highest value of 9 ± 1 in F12-K + protein (unpaired *t*-test, *p* value < 0.001). However, at 400 mV/s, lower *I*_pa_/*I*_pc_ values were observed in PBS as compared to those at 50 mV/s, which indicates the involvement of electrochemical fouling at a slow scan rate. Subsequently, we examined the *I*_pa_/*I*_pc_ value at the higher scan rate (i.e., 400 mV/s) in F12-K and F12-K + protein. Interestingly, the *I*_pa_/*I*_pc_ value was found to be lower at 400 mV/s for all electrodes (Table 4). At 400 mV/s, the *I*_pa_/*I*_pc_ values for PyC were higher than those for MWCNT/ta-C and CNF/ta-C, indicating that lesser DAQ is available to the surface, which is possibly due to the blockage of adsorption sites in the presence of biofouling components (Table 4). The difference in *I*_pa_/*I*_pc_ values between 50 and 400 mV/s scan rates was higher in F12-K + protein than that in F12-K. The CNF/ta-C electrode showed the lowest *I*_pa_/*I*_pc_ difference in F12-K (14%), whereas in F12-K + protein, MWCNT/ta-C and CNF/ta-C showed approximately similar drop rates of 42 and 43%, respectively. Note that the PyC electrode also exhibited the highest difference in the *I*_pa_/*I*_pc_ values between 400 and 50 mV/s in the two biological media (65% in F12-K and 71% in F12-K + protein). This indicates that the presence of biofouling components, in combination with electrochemical fouling at 50 mV/s, has the most significant effect on the PyC surface, thus shrinking the reduction peak of DAQ ⇀ DA at slow scan rates.

**Figure 5:**
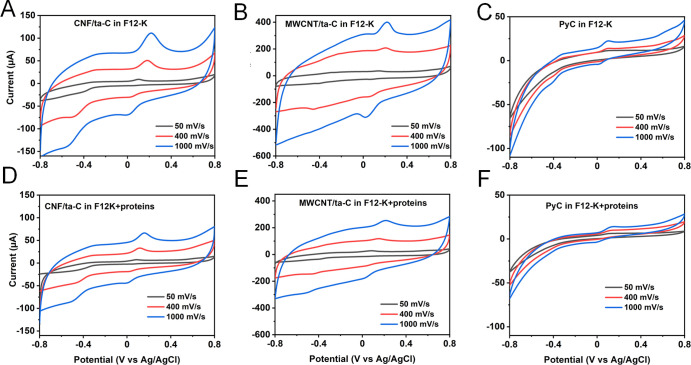
CVs of DA reactions for (A,D) CNF/ta-C, (B,E) MWCNT/ta-C, and (C,F) PyC at different scan rates in F12-K and F12-K + protein. The concentration of DA is 100 μM.

We compared the peaks obtained due to the LDAC ⇋ DAC redox couple in F12-K and F12-K + proteins at different scan rates in order to gain insights into electrochemical fouling in F12-K + protein (Figure 5). In the case of CNF/ta-C, a broad peak due to LDAC ⇀ DAC (around −0.3 V) was observed at all scan rates, while the DAC ⇀ LDAC peak (around −0.5 V) was prominent in both media. Similar behavior was demonstrated by MWCNT/ta-C, where DAC ⇀ LDAC formed a noticeable peak, whereas LDAC ⇀ DAC formed a broad (almost invisible) peak. For CNF/ta-C and MWCNT/ta-C electrodes, the peaks associated with DA ⇋ DAQ shifted cathodically in F12-K + protein in comparison to F12-K. Conversely, a small anodic shift for PyC in F12-K + protein was observed in comparison to F12-K. In contrast to F12-K, the peaks associated with LDAC ⇋ DAC were unnoticeable on the PyC surface in F12-K + protein. This indicates that the availability of PyC for LDAC ⇋ DAC reactions in F12-K + protein is worse than that of CNF/ta-C and MWCNT/ta-C.

To further estimate the influence of proteins and biological media on the electron-transfer kinetics and electrochemically active areas of the electrodes, we maintained the electrodes at a positive potential of 0.5 V for 2 min in each cycle (Figure S6). The positively charged electrode surface was expected to first attract the negatively charged proteins effectively and cause the adsorption of the proteins on the surface. Second, during the 2 min of the holding potential, DOQ formed after the oxidation could also complete its chemical reaction step, resulting in no (or very small) DOQ ⇀ DA reduction peak during the reverse scan. Holding at a positive potential for CNF/ta-C in F12-K, an anodic shift was observed in the oxidation peak of DA, and the LDAC ⇀ DAC peak became more prominent because of the holding procedure, as expected. In F12-K + protein, a drop in the current with the proceeding cycling was observed, indicating that the electrochemically active area decreased owing to the adsorption of the proteins. Moreover, MWCNT/ta-C showed a greater current drop in F12-K than that in F12-K + protein with increased cycling, which corresponds to the results discussed above. However, the PyC surface was completely fouled in F12-K and F12-K + protein, as indicated by the complete disappearance of DA ⇋ DOQ peaks after the holding potential. The surface passivation during the holding time of 2 min in each cycle affected the DA electrochemistry on all electrodes, yet the most severe effects were observed on PyC.

### Recovery from Biofouling

**Figure 6:**
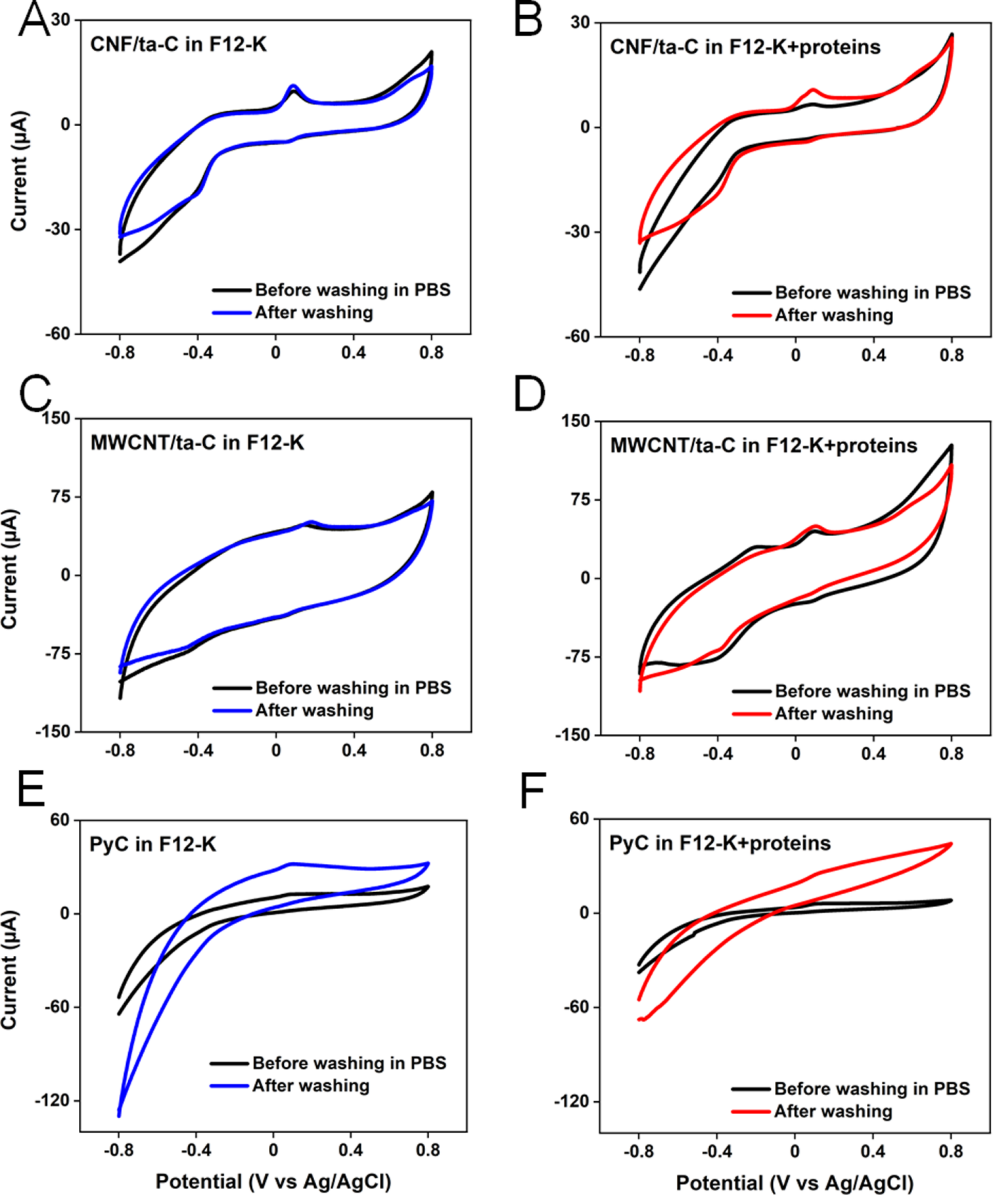
CVs of (A,B) CNF/ta-C, (C,D) MWCNT/ta-C, and (E,F) PyC in F12-K and F12-K + protein before and after PBS washing at 50 mV/s for 10 cycles.

We examined the effect of PBS washing on the performance of the electrode surfaces after they exhibited fouling tendencies (Figure 6). The electrodes were washed in PBS for 10 cycles at 50 mV/s. Washing CNF/ta-C in PBS after being cycled in F12-K showed similar peak currents of DA and shape of CV, indicating that PBS washing did not significantly improve the electrode performance. The CNF/ta-C electrode fouled in F12-K + protein showed prominent oxidation and reduction peaks of both redox couples after PBS washing, with the retention of LDAC ⇋ DAC peaks indicating that the proteins most likely bonded to the electrode surface through secondary interactions only. The MWCNT/ta-C electrode in F12-K showed the same behavior before and after PBS washing, with a slight increase in oxidation current. The CV of MWCNT/ta-C in F12-K + protein after PBS washing showed an increase in DA oxidation current, but the reduction peak of DOQ ⇀ DA and the oxidation peak of LDAC ⇀ DAC almost vanished with a decrease in the double-layer current. This indicates that the proteins are somewhat difficult to remove from this electrode, which is attributable to their physical trapping into a porous-network-type structure. The fouled PyC in F12-K + protein did not show any improvement after PBS washing. Both oxidation and reduction peaks disappeared, with the CV shape indicating an irreversible reaction. As the geometrical features were absent in this case, this strongly indicates that the interaction between the proteins and PyC was stronger than that for the other two electrodes. To summarize, a comparison of MWCNTs in F12-K and F12-K + protein after PBS washing indicates that the washing did not recover the electrode performance, whereas the CNF/ta-C electrode in F12-K + protein performed better after being washed.

## Conclusions

We evaluated the effects of electrochemical fouling (in PBS), biofouling (in cell-culture media F12-K with and without protein addition), and their combination on different nanocarbon electrodes with different surface morphologies. The SEM, XAS results, and Raman spectra showed that the CNF/ta-C electrode exhibits forest-like geometry with numerous functional groups, such as carboxyl and carbonyl groups, and is defective. The MWCNT/ta-C electrode exhibits spaghetti-like geometry with a porous structure and is highly defective. In contrast, PyC exhibits a smooth surface containing sp^3^- and sp-type carbon and negligible functional groups. The contact angle measurements showed that the CNF/ta-C and MWCNT/ta-C electrodes were less wettable than the PyC electrode. In addition, the electroanalytical experiments conducted using an OSR probe, [Ru(NH_3_)_6_]^3+^, showed that biofouling was not extensive enough to affect the OSR probe. The biological media showed similar effects on all electrodes regardless of their different geometries. In addition, semi-infinite diffusion was the major factor contributing to the mass transfer in all electrodes. Reversible electron-transfer kinetics were observed for CNF/ta-C and PyC, whereas MWCNT/ta-C exhibited quasi reversible kinetics, as indicated by the Matsuda–Ayabe parameter. While cycling from the 1st to 10th cycle at 50 mV/s, the fouling elements reduced the electrochemically active areas for all electrodes, with no noticeable effect on DA reaction kinetics. In contrast to planar PyC, the CNF/ta-C electrode showed a lower extent of electrochemical fouling and faster electron-transfer kinetics; these characteristics are comparable to those of the MWCNT/ta-C electrode. The presence of biological components blocked the DA adsorption sites, resulting in a smaller oxidation current in comparison to the measurements conducted in PBS. The difference in the *I*_pa_/*I*_pc_ values between the scan rates of 400 mV/s (partial outrunning of the DAQ ⇀ DAC step) and 50 mV/s (formation of DA fouling components) showed that in the presence of the biological media, the electrode surfaces exhibited rapid passivation due to the electrochemical fouling effect in addition to biofouling at a slower scan rate. Of the three electrodes studied, planar PyC, containing highly reactive sp-type carbon, exhibited the highest passivation tendency. In F12-K + protein, hydrophobic MWCNT/ta-C and weakly hydrophilic CNF/ta-C (possessing considerable functionalities) exhibited similar passivation, as demonstrated by the decreases in the values of *I*_pa_, Δ*E*_p_, and *I*_pa_/*I*_pc_. An adsorption-controlled reaction for DA was predominantly observed on MWCNT/ta-C in the biological media. Washing the electrodes in PBS for 10 cycles at 50 mV/s after being fouled in the biological media could only recover the performance of the CNF/ta-C electrode. In conclusion, rapid surface passivation occurs in the presence of biological media due to the combined effect of biofouling and electrochemical fouling. Electrodes containing composite nanostructures showed decreased biofouling and electrochemical fouling than the planar electrode, as indicated by the faster reaction kinetics and a lower current drop.

## Materials and Methods

### Material Fabrication

#### ta-C Fabrication

A 20 nm thick Ti layer was deposited on the surface of a highly conductive (0.001–0.002 Ω cm) p-type Si wafer (Utrasil) using direct current–magnetron sputtering (DC–MS). Filtered cathodic vacuum arc (FCVA) was used for the deposition of ta-C on top of the Ti adhesion layer, without breaking the vacuum in between. Then, a 2-in Ti target (Kurt J. Lesker Company) at a deposition distance of 220 mm from the sample was installed for DC–MS. During the deposition, a discharge power of 100 W, total pressure of 0.67 Pa, and an Ar gas flow rate of 29 sccm were applied. FCVA was installed with a dual cathode configuration with two graphite cathodes (6.35 mm diameter, 99.95% purity, Goodfellow). A capacitor bank of 2.6 mF was charged to 400 V, maintaining an arc current of 0.7 kA and pulse width of 6 ms. During the deposition, the total pressure was maintained below 10^–4^ Pa.^18^

#### CNF/ta-C Fabrication

CNFs were grown on ta-C using plasma-enhanced chemical vapor deposition (CVD). Cathodic arc deposition was used to deposit a 10 nm thick Ni layer on the ta-C surface. The catalyst-coated ta-C wafers were annealed for 3 min at 400 °C in a cold-wall CVD reactor at a chamber pressure of <10^–2^ mbar. The chamber was heated to 400 °C at a ramp speed of 250 °C/min. After the annealing step, the chamber pressure was increased to 0.1 m bar, and NH_3_ buffer (100 sccm) was used to fill the chamber. Then, the chamber temperature was increased to 750 °C at a ramp speed of 300 °C/min. Next, 150 W DC plasma was ignited in the chamber by injecting a carbon precursor C_2_H_2_ (30 sccm) and increasing the NH_3_ flow to 125 sccm after the chamber temperature reached 675 °C. The growth phase continued for 30 min, producing vertically aligned fibers.^19^

#### MWCNT/ta-C

Catalyst layers of 0.2 nm Al, 2 nm Co, and 2 nm Fe were coated on the ta-C wafers by radio frequency sputtering and e-beam evaporation. MWCNTs with curved and partially tangled geometry were grown directly on the catalyst-coated ta-C wafers. The samples were heated to 550 °C using an electrically heated graphite holder in a low-pressure CVD reactor at 10 m bar pressure for 10 min to reduce the catalyst metals. The reactor chamber was subjected to evacuation, and a N_2_ buffer gas at 250 sccm concentration was used to fill the chamber under 10 m bar process pressure. To synthesize MWCNTs on the catalyst-coated ta-C wafer surface, a carbon precursor (C_2_H_2_) with 25 sccm concentration was introduced into the chamber for 10 min at a constant temperature of 550 °C.^23^

#### Pyrolytic Carbon

To deposit a thin carbon film on top of the silicon wafer, pyrolysis was conducted. To remove the native silicon oxide layer from the Si wafer and make it more hydrophobic, a 4-in Si wafer was dipped into a 10:1 deionized water and hydrofluoric acid solution. A 10 mm thick layer of a spin-coated negative photoactive polymer (photoresist) SU-8 50 (MicroChem) was deposited on top of the silicon wafer using a BLE spinner (Georgia Tech) at 9000 rpm for 45 s. The resist was soft-baked on a hotplate using the standard protocol [flood exposure for 8 s at a 365 nm wavelength using a mask aligner (Sûss MicroTec)], followed by baking on the hotplate. The wafers were diced into 10 cm × 10 cm pieces using a Loadpoint MicroAce Series 3 dicing saw.

The pyrolysis process was performed in a Nabertherm RS 170/1000/13 horizontal tube furnace. Nitrogen gas was flushed into the tube three times under vacuum in order to remove the oxygen gas present inside the furnace. After the last flush, a low nitrogen flow and ambient pressure were maintained inside the tube. The furnace was first heated to 300 °C and held at this temperature for 40 min to remove the remaining oxygen from the film. Then, the temperature was increased to 900 °C and maintained for 60 min to facilitate the pyrolysis process. Then, the furnace was gradually (for 12 h) cooled to room temperature.^16^

### Characterization of Samples

The morphology of the samples was studied using a scanning electron microscope (JEOL JSM-7500FA). Raman spectroscopy was performed using a Micro-Raman spectroscope (WITec Alpha 300 RA+) equipped with an optical microscope. The measurements were performed at a laser excitation wavelength of 532 nm using a 50× objective lens. Line scanning was performed using a 3 mm line length containing 50 points (10 accumulations per point) and 0.5 s integration time. The Raman data were fitted with Lorentzian curves.

Contact angle measurements were performed using optical goniometry (contact angle meter theta, Biolin Scientifics). Advanced and receding contact angles were measured using the sessile droplet method. For measuring the advanced contact angle, we used 2–8 μL of water droplets with an increasing rate of 0.06 μL/s, whereas for measuring the receding contact angle, we decreased 8 μL of water droplets to 0 μL at a rate of 0.16 μL/s.

### Electrochemistry

Cyclic voltammetry was performed using a Gamry Reference 600 potentiostat with a three-electrode setup containing a Ag/AgCl reference electrode and a platinum wire as the counter electrode. The reference electrode, formed of a silver wire coated with AgCl, was used for performing the measurements in a biological environment. The solutions were purged with nitrogen gas for 15–20 min. The pH value was checked and adjusted to 7.4 for F12-K Gibco Nut Mix with and without proteins (15% HS, 2.5% FBS, and 1% penicillin streptomycin) using a 2 M HCl solution. Fresh electrodes were prepared for each measurement. The uncompensated resistance (*R*_u_) of each electrode was measured in PBS. The PBS solution was prepared by mixing 8 g of NaCl, 0.2 g of KCl, 1.44 g of Na_2_HPO_4_, and 0.24 g of KH_2_PO_4_ in 800 mL of distilled water. The pH of the solution was maintained at 7.4 using a 1 M HCl or NaOH solution. The total volume of the solution was increased to 1 L by adding more distilled water.

The samples were connected to a copper FR4-PBB sheet from the backside after their surfaces were exfoliated using a diamond and copper palette. The surfaces were masked using a polytetrafluoroethylene (PTFE) tape. A 3 mm diameter hole was formed on the PTFE tape surface to define the geometric surface area of the electrodes.

DA chloride and Ru(NH_3_)_6_Cl were purchased from Sigma-Aldrich. F12-K Gibco Nut Mix, HS, FBS, and penicillin streptomycin were obtained from Fisher Scientific. The 1 mM Ru(NH_3_)_6_Cl (OSR probe) and 100 μM DA solution (ISR probe) in PBS; biological medium (F12-K Gibco Nut Mix); and biological medium with 2.5% FBS, 15% HS, and 1% penicillin streptomycin were evaluated separately. The effects of the proteins and biological medium (F12-K) components on [Ru(NH_3_)_6_]^3+^ and DA detection were investigated using the scan rate ranging from 5 V/s to 10 mV/s. All measurements were performed at room temperature. The average values of all parameters with standard deviations obtained for 2–4 samples were calculated. One-way ANOVA or *t*-test were performed to evaluate the significance of the differences among results. Statistical *p* values were considered significant at the 95% confidence interval (*p* < 0.05)..

## Supporting Information Available

Additional details of calculations of parameters, SEM images, background measurements, and additional experimental results (PDF)
