# Supplementary material for: Nanostructured Geometries Strongly Affect Fouling of Carbon Electrodes
Source: ACS Omega. 2021 Sep 29;6(40):26391–403. doi: 10.1021/acsomega.1c03666 (PMC8515610; doi:10.1021/acsomega.1c03666)
Supplement: Supplementary file 1 — ao1c03666_si_001.pdf [file ao1c03666_si_001.pdf]

Supporting Information

Nanostructured geometries strongly affect fouling of carbon electrodes

Ayesha Kousar [†], *Emilia Peltola* [†], *Tomi Laurila* [†‡] [*]

[†]Department of Electrical Engineering and Automation, School of Electrical Engineering, Aalto University, 02150 Espoo, Finland.

[‡]Department of Chemistry and Materials Science, School of Chemical Engineering, Aalto University, 02150 Espoo, Finland.

Corresponding Author:

* Email: tomi.laurila@aalto.fi

Calculation of heterogeneous electron transfer rate constant (k_0)

Standard rate constant was calculated using Nicholon's method. The relationship between standard rate constant and Nicholson's dimensionless number is given by following equation.

$$k_0 = [\frac{(\pi.D_o.f.v)^{\frac{1}{2}}}{(D_o/D_R)^{\frac{\alpha}{2}}})].\Psi$$

Where k_0 is heterogeneous electron transfer rate constant, Ψ is the Nicolson's dimensionless number dependent on ΔE_p[1]. D_o is the diffusion coefficient of oxidative specie while D_R is the coefficient of reductive specie for $[Ru(NH_3)_6{}^{+3}]$ redox reaction. D_o and D_R was taken to be 5.47×10^{-6} and 7.70×10^{-6} $cm^2\ s^{-1}$ from already published report[2]. v is the scan rate in V s^{-1}, α is the charge transfer coefficient and f = (nF/RT) in which n is number of electrons tranferred during the redox process, F is Faraday constant with the value of 96485 C mol^{-1}, R is the ideal gas constant with the value of 8.314 J mol K^{-1} and T is the temperature in K.

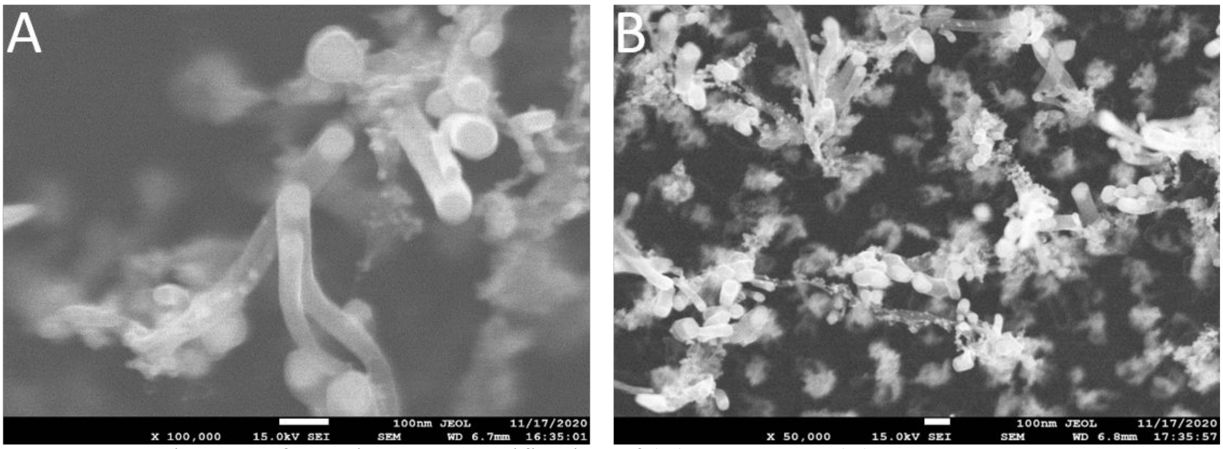

Figure S1. SEM images of CNF/ta-C at magnification of (A) 100,000 X, (B) 50,000 X

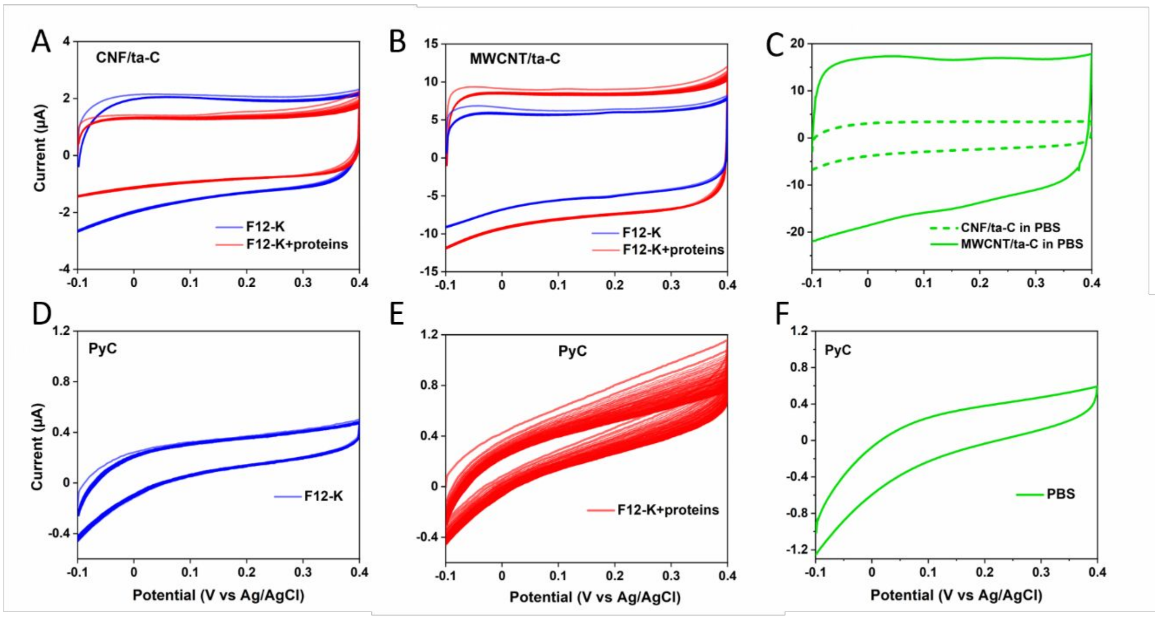

Figure S2. Cyclic voltammograms showing the double layer current for (A) CNF/ta-C and (B) MWCNT/ta-C in F12-K and F12K+proteins (50 cycles) (C) CNF/ta-C and MWCNT/ta-C in PBS, (D) PyC in F12-K, (E) in F12-K+proteins (50 cycles) and, (F) in PBS.

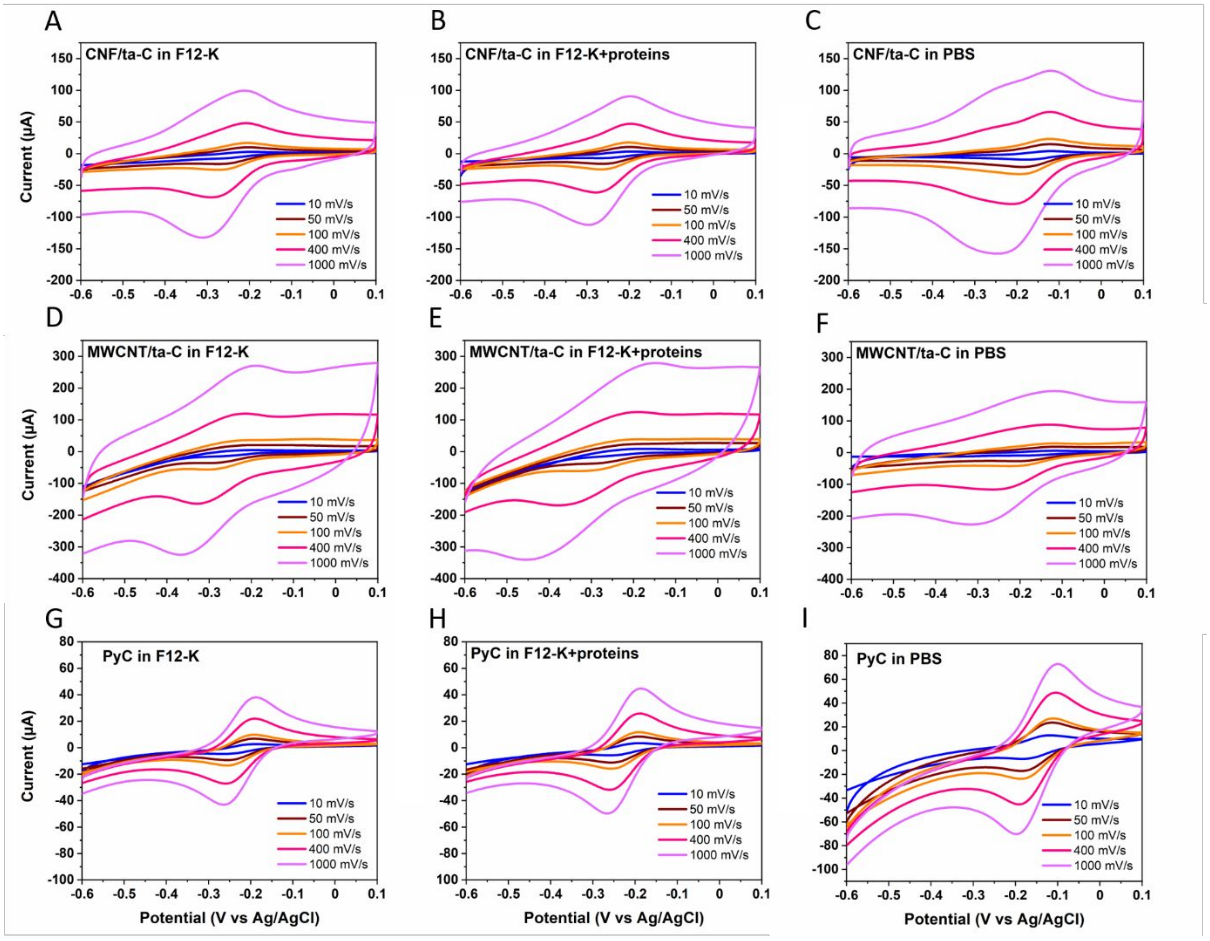

Figure S3. Electrochemistry of [Ru(NH$_3$)$_6$$^{+3}$] for (A, B, C) CNF/ta-C, (D, E, F) MWCNT/ta-C and (G, H, I) PyC at different scan rates in F12-K, F12-K+proteins and PBS.

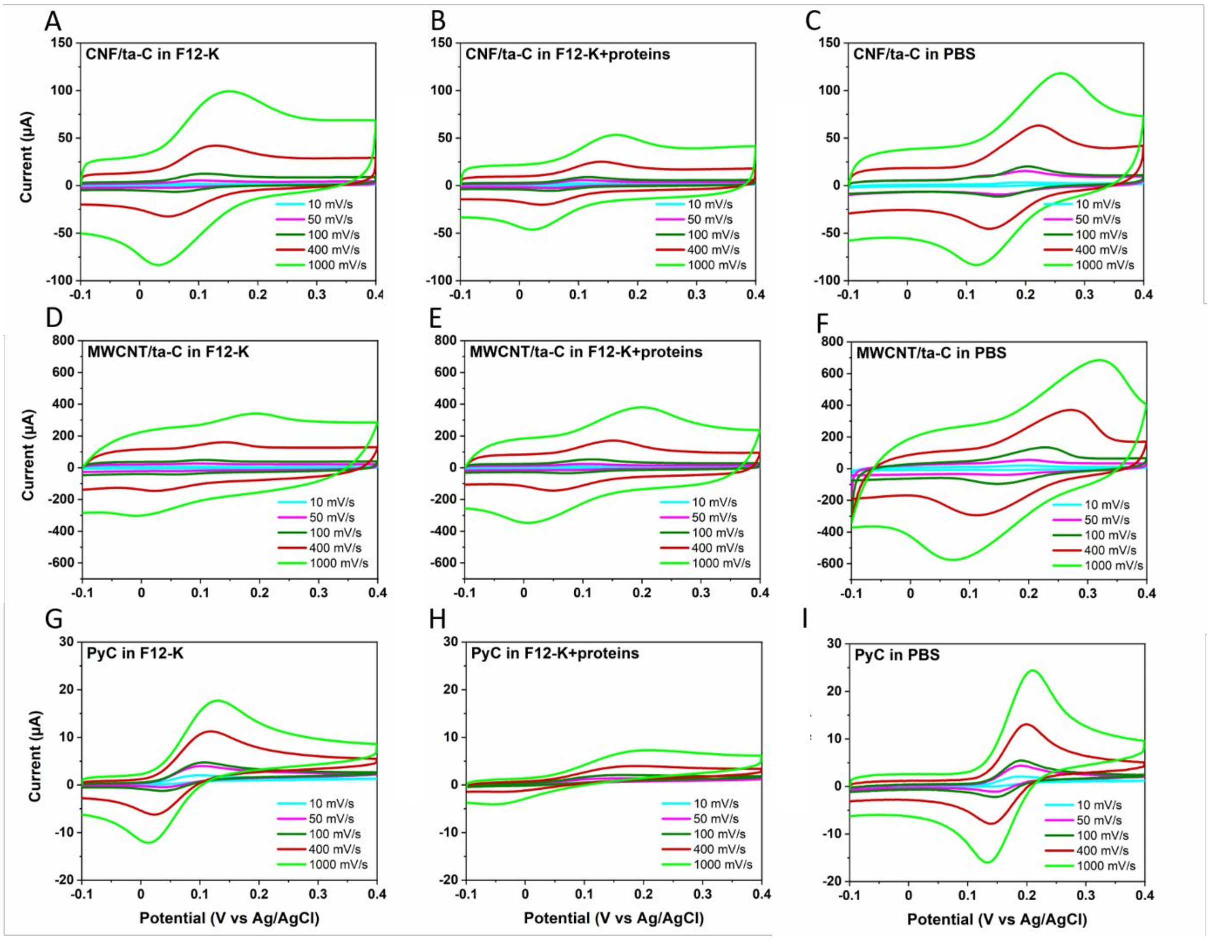

Figure S4. Electrochemistry of Dopamine for (A, B, C) CNF/ta-C, (D, E, F) MWCNT/ta-C and (G, H, I) PyC at different scan rates in F12-K, F12-K+proteins and PBS.

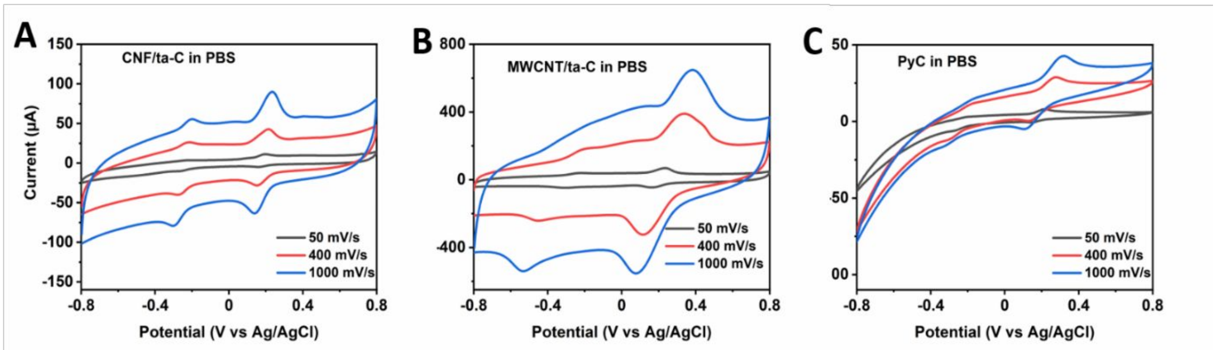

Figure S5. Cyclic voltammograms of DA reactions for (A) CNF/ta-C, (B) MWCNT/ta-C and (C) PyC at different scan rates in PBS.

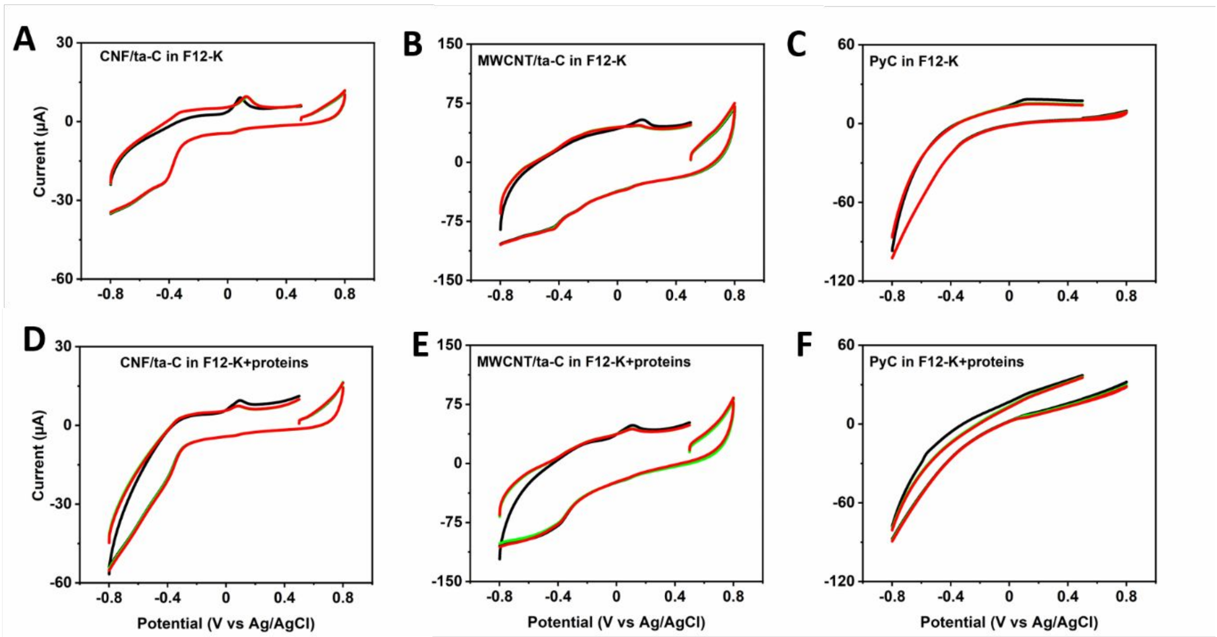

Figure S6. Holding potential experiments at 0.5 V for two minutes in each cycle at 50mV/s for (A, D) CNF/ta-C, (B, E) MWCNT/ta-C and (C, F) PyC in F12-K and F12K+proteins. DA concentration is 100 μM. Ascending order for proceeding cycles is denoted by black, red and green colour.

References

(1) Nicholson, R. S. Theory and Application of Cyclic Voltammetry for Measurement of Electrode Reaction Kinetics. *Anal. Chem.* **1965**, *37* (11), 1351–1355.

(2) Wang, Y.; Limon-Petersen, J. G.; Compton, R. G. Measurement of the Diffusion Coefficients of [Ru (NH3) 6] 3+ and [Ru (NH3) 6] 2+ in Aqueous Solution Using Microelectrode Double Potential Step Chronoamperometry. *J. Electroanal. Chem.* **2011**, *652* (1–2), 13–17.
